# Pennsylvania State Dental Society

**Published:** 1884-12

**Authors:** W. H. Trueman


### PENNSYLVANIA STATE DENTAL SOCIETY.

### SIXTEENTH ANNUAL MEETING, HELD AT WILKESBARRE, JULY 29, 30 AND 31, 1884.

REPORTED FOR THE INDEPENDENT PRACTITIONER BY W. H. TRUEMAN, D. D. S.

(Continued from page 641.)

### EVENING SESSION.

Dr. Henry Leffman, of Philadelphia, read a paper, entitled "Reflections on the Practical Bearing of the Germ Theories of Disease."

The germ theory of disease has probably had advocates ever since the time that the microscope enabled investigators to detect the minute forms of life, long before the present methods of culture and illumination and staining placed in our hands the power of differentiation much greater than mere enlargement; but it is only within the last few years that a series of demonstrations, thoroughly logical in character, have been presented.

A dental society would have little practical interest in these questions if it were not for the fact that a strong disposition has been shown of late to ascribe to micro-organisms a causative relation to dental caries. It is, of course, perfectly natural that such a theory should be advanced. An exceedingly common and serious affection, occurring under all conditions, the exciting cause not known, it would naturally follow that a new and attractive theory of disease should be extended to such an affection, especially as the microscope reveals a variety of organisms in and around the cavity of decay. There is no doubt that the germ theory of disease is becoming more generally accepted; in relation to some diseases the doctrine has been established for years, but these were rare diseases; malignant pustule for instance. No one who investigates this disease seems to doubt its bacillar origin. To discover the practical bearings of the theory we must turn to diseases more general and of greater importance, as for instance the bacillar origin of phthisis. During the past three years the discoveries of Robert Koch have been subjected to the closest scrutiny, dictated only by a scientific spirit, but undoubtedly biased by strong unbelief and personal opposition. Every demonstration, method, and inference Koch has put forth has been attacked, and efforts have been made to give them other significance than that he has assigned, yet his position appears to be unshaken, and his followers increase daily. However unwelcome the truth, I think we may feel assured that the tendency of opinion is to the view that consumption is due to micro-organisms, which are reproduced in the affected tissues.

Now let us see what are the practical bearings of this doctrine, and how far they are applicable to the microbe theory of caries. It is generally admitted that there must be a predisposition; this is essential. By this is meant a condition of the system, local or general, which fits it to receive and become the prey of micro-organisms. A paper has lately appeared, by Arthur S. Underwood, lecturer on dental anatomy and physiology at the Dental Hospital of the London Medical School, in which the microbe theory of dental caries is very strongly advocated. He remarks that the initial stage, the destruction of the enamel, is due to an acid secretion, the result of a vegetable growth. A number of experiments are detailed, which go to show that decomposing animal or vegetable matter containing organic acids is incapable of producing marked change in tooth structure, unless under conditions in which micro-organisms can be developed. We must remember the experiments were made upon teeth out of the mouth, and under conditions not found in the mouth; this detracts very much from their practical value. The point, however, to which I desire to call especial attention is this: even if we admit the agency of micro-organisms, the practical bearing of such doctrine is slight. In this disease, as in all others, the result is due largely to predisposition. It is well established that dental decay is not purely traumatic, but that susceptibility to it is governed by systemic conditions. Original predisposition may govern its greater or less extent, and while we may agree that the real process is local and external, yet this local and external action is preceded by a local reduction of the vital resisting power of the tooth structure. The doctor referred to various diseases of fruit trees and of plants, such as mildew, the various blights, etc., as examples of apparently localized pathological changes, purely traumatic in character, yet in all cases, as shown by careful experiment, there is first a depressed vitality which renders the plant susceptible to external agents.

In conclusion, he held that the demonstration of micro-organisms as a cause of dental caries does not add anything practically to the means of prevention and treatment of the malady, over and above that which clinical observation has taught. If it leads as similar views in general medicine are now leading, to exaggerated confidence in antiseptics and germicides, it will do more harm than good.

*Dr. Pierce*—Referred to the three theories advanced to account for dental caries, known as the chemical, the pathological, and lastly the germ theory, and questioned if either could be considered a full explanation of their cause. The destructive action seemed a complex one, and probably at some stage of the process each contributed to the result. The later investigations have been conducted so carefully, especially those of Dr. Miller, and they seem to confirm previous observations so markedly, that they deserve very careful consideration. It is too soon, perhaps, to estimate their practical value, or what effect they may have upon our practice. We cannot know too much of the subject; the more we know of the cause and progress of a disease, the better will we be able to treat it. He thought that in the future, effort will be directed more towards prevention than to improvements in methods or materials for filling. He gave a history of the theory that decay was due entirely or in part to minute forms of vegetable or animal life, from the time that a German writer first directed attention to it some twenty years ago. Then, as now, the destruction was supposed to be caused by vegetable growths, a number of forms of which are always to be found in the oral cavity, and especially in the cavity of decay. He referred to a paper he had published some years ago, in which these forms were illustrated and described. It has been a mooted question whether they are the cause of decay, or merely thrive in its presence; whether they are the real disintegrating agents, or whether their presence so changes the secretions that they become destructive. If the recently published statements of Miller, Underwood, and others are confirmed, the latter idea seems more probable.

*Dr. C. S. Beck*—Spoke of his observations in the winter cultivation of greenhouse plants, and the constant watchfulness necessary to guard them from the various diseases and pests Dr. Leffman had referred to. In the greenhouse the plants were in an unnatural condition. It was a forced growth, out of season, and in what is really to them an unnatural atmosphere; in consequence their vitality is low; this is a predisposing cause. The exciting cause is always present. As long as the plants are kept in vigorous health there is no trouble; but if from any cause their vitality is still further reduced, either from excessive dryness or excessive moisture, too much or too little heat, etc., even if this condition exists but for a few minutes, within a short time the disease attacks every part of the plant that has suffered. Growing naturally, they may be subjected to far greater changes without this result following. So with our bodies and with our teeth; the active causes of disease are always present, but are powerless for harm until, either generally or locally, a depressed vitality acts as a predisposing cause. He had been much interested in watching the gradual development of this theory, and hoped that as it becomes better understood, we may be able to succeed as well in preventing decay as we now succeed in filling the cavities it has made.

*Dr. Gerhart*—Suggested that if the idea of Dr. Koch was correct, there was little use for quarantine; if it was essential that there must be a reduced vitality, and the disease germs are always present, what does quarantine regulations accomplish? Either he did not understand the germ idea, or he could not accept it. He noticed when the potato rot was epidemic in this country, some twenty-eight years ago, that it affected the finest and largest potatoes first and most severely; their size seemed evidence of vigor and healthy growth, yet they suffered most.

*Dr. Leffman*—Replied that they, being more highly cultivated and farther removed from the original type, were of lower vitality, and naturally offered less resistance to the disease.

*Dr. Pierce*—Remembered the potato rot epidemic referred to by Dr. Gerhart, and said he had no doubt it was due entirely to the peculiar and unusual climatic influence of that year. The spring and early summer had been dry and cold, and very unfavorable to vegetation. This was followed by frequent rains, and a moist, warm atmosphere, or, as the farmers say, "good growing weather." Vegetation of all kinds began to grow with great rapidity, and this condition naturally produced a low degree of vitality that was unable to resist the disease germs. The fact that the potato rot was so prevalent and destructive that year, was no evidence that the disease germs were more numerous or more active; they are always present, and every year some potatoes rot, but not to so great an extent as to attract attention. It is the same with the rust on wheat. If climatic conditions favor a low vitality in the plants, they are predisposed to its attack, and we have general complaint of the loss it occasions; but ordinarily so few plants suffer that it is not noticed. That diseases are more malignant and unmanageable during epidemics, is due to the same cause. The climatic conditions may be unhealthy, people become excited, and from various causes are less able to resist the disease. The theory is not at all new, but the introduction of purely scientific methods of study and research is so recent, and has opened so wide a field to investigation, that we can hardly see to what it may not lead. The tendency at present in all branches of medicine is largely to prevention, and the result is seen in the fact that diseases that formerly as epidemics extended over large areas and almost depopulated cities, are now far less frequent and fatal, and are usually confined to the locality in which they are developed.

*Dr. William H. Trueman*—Considered the subject presented of great and, in the light of recent investigations, of increasing importance. The germ theory explains many pathological conditions that have long been mysterious, and in surgery, especially, its adoption has been followed by remarkable results. Operations that formerly were considered extremely hazardous are now performed with comparative safety; and so generally is it accepted, and the beneficial results of the precautions it suggests so widely recognized, that few surgeons attempt even trifling operations without adopting them. It may be that harm has been done by depending upon antiseptic methods to the neglect of other equally important precautions, but that is no fault of the theory. The recognition of the baleful work of these infinitesimal, omnipresent germs, and the adoption of means to either destroy or exclude them, has been a wonderful advance in all departments of medicine. Now, if we can use in the mouth some safe and efficient germicide, may we not hope by that means to check the ravages of decay? If we can use in the cavity some agent that shall prevent them from again entering it, that may assist very materially in making our present methods of filling more permanent and useful. In fact, the preservative properties some filling materials possess is probably owing in a great measure to the salts formed by their oxidation. The possibilities suggested by this theory are well worth a careful and earnest study.

The morning session of Wednesday, July 30, was omitted, to allow those members, who desired, to participate in a visit to a coal mine, arranged by Dr. C. S. Beck. About thirty, including a number of ladies, descended Hollingbrook Mine, No. 2, in charge of Supt. Smythe, who proved an interesting and instructive guide. This mine was selected on account of its freedom from gas, thus permitting the use of open lights, and also as, being remarkable dry, it could be explored with comfort, at the same time giving an excellent idea of the various operations of mining and preparing coal for the market. After the gentlemen were provided with lights, all stepped upon the elevator and were gently lowered about six hundred and forty feet. The galleries we found high, wide, and well ventilated, and as the visitors walked along, listening to Mr Smythe's description of the various methods of mining, it was hard to realize that they were so far removed from the busy, active world above; and the information that they had passed under a wide river, and were at that time about nine hundred feet beneath the town of Wilkes Barre, was received with surprise. The dangers of mining were illustrated by one of the workmen to whom our attention was called, who, with five companions, had suffered from an explosion of gas, and he alone survived. The scars on his hands and face showed how severely he had been burned, and how much he had suffered. Although the utmost care is used, such accidents occasionally occur, and are frequently the cause of severe loss of life, and of great suffering. After a long tramp all retraced their steps to the shaft, and were "elevated" without accident or mishap, having thoroughly enjoyed the visit.

(TO BE CONCLUDED.)

